# Vitamin D in Melanoma: Potential Role of Cytochrome P450 Enzymes

**DOI:** 10.3390/life14040510

**Published:** 2024-04-15

**Authors:** Mohamed Ben-Eltriki, Erysa J. Gayle, Jhoanne M. Paras, Louisa Nyame-Addo, Manik Chhabra, Subrata Deb

**Affiliations:** 1 Clinical Pharmacology Lab, Department of Pharmacology and Therapeutics, Max Rady College of Medicine, University of Manitoba, Winnipeg, MB R3E 0T6, Canada; 2 Cochrane Hypertension Review Group, Therapeutic Initiative, University of British Columbia, Vancouver, BC V6T 1Z3, Canada; 3 College of Biomedical Sciences, Larkin University, Miami, FL 33169, USA; egayle@myularkin.org (E.J.G.); jparas@myularkin.org (J.M.P.);; 4 Department of Pharmaceutical Sciences, College of Pharmacy, Larkin University, Miami, FL 33169, USA

**Keywords:** vitamin D, melanoma, cytochrome P450, therapeutic prevention, dermal expression, anticancer effects, supplementation

## Abstract

Vitamin D is a promising anticancer agent for the prevention and treatment of several cancers, including melanoma. Low 25-hydroxyvitamin D levels, a routinely used marker for vitamin D, have been suggested as one of the factors in the development and progression of melanoma. The parent vitamin D needs activation by cytochrome P450 (CYP) enzymes to exert its actions via the vitamin D receptor (VDR). This review discusses the role of vitamin D in melanoma and how CYP-mediated metabolism can potentially affect the actions of vitamin D. Through interacting with the retinoid X receptor, VDR signaling leads to anti-inflammatory, antioxidative, and anticancer actions. Calcitriol, the dihydroxylated form of vitamin D_3_, is the most active and potent ligand of VDR. CYP27A1, CYP27B1, and CYP2R1 are involved in the activation of vitamin D, whereas CYP24A1 and CYP3A4 are responsible for the degradation of the active vitamin D. CYP24A1, the primary catabolic enzyme of calcitriol, is overexpressed in melanoma tissues and cells. Several drug classes and natural health products can modulate vitamin D-related CYP enzymes and eventually cause lower levels of vitamin D and its active metabolites in tissues. Although the role of vitamin D in the development of melanoma is yet to be fully elucidated, it has been proposed that melanoma prevention may be significantly aided by increased vitamin D signaling. Furthermore, selective targeting of the catabolic enzymes responsible for vitamin D degradation could be a plausible strategy in melanoma therapy. Vitamin D signaling can be improved by utilizing dietary supplements or by modulating CYP metabolism. A positive association exists between the intake of vitamin D supplements and improved prognosis for melanoma patients. Further investigation is required to determine the function of vitamin D supplementation and specific enzyme targeting in the prevention of melanoma.

## 1. Background

Melanoma is the fifth most frequently diagnosed malignancy type in both genders in the United States and is considered one of the most fatal cancer types, especially after metastasis [1,2]. The incidence rates are much higher in other parts of the world, such as Europe and Australia. Melanoma accounts for only 2.3% of skin cancer cases; however, it is responsible for 75% of skin cancer-related deaths, making it one of the most perilous types of skin cancer [3]. With UV radiation exposure being the main environmental factor, family history of melanoma, altered regulation of susceptibility genes, weight, and hair and skin color are among some of the risk factors that epidemiological studies have discovered [4,5,6]. When it comes to skin cancer, people with darker skin types are twenty times less likely to get it than lighter-skinned people living at the same latitude [4]. The prognosis of melanoma drops drastically once it starts to metastasize [2]. Aside from genetic and environmental risk factors, the expression and regulation of genes involved in the anticancer protective effects of melanoma can be a crucial factor in the development and progression of melanoma [4]. 

Vitamin D, a lipid-soluble vitamin, can be obtained from animals (vitamin D_3_ or cholecalciferol) or plants (vitamin D_2_ or ergocalciferol). Cholecalciferol, or vitamin D_3_, is formed in the skin from 7-dehydrocholesterol (7-DHC) through UV exposure and is the natural form of vitamin D [7,8]. The synthesis of vitamin D can vary based on the season and length of daytime sunlight, the time of day, weather, usage of sunscreen, and the amount of melanin found in the skin [9]. Vitamin D can be obtained from dietary components, including fatty fish (e.g., mackerel, salmon, and tuna), mushrooms, fish liver oils, egg yolks, red meat, and cheese [10,11,12,13]. The endogenous vitamin D_3_ and exogenous vitamin D_2_/D_3_ get absorbed via simple diffusion and intestinal membrane carrier proteins, and subsequently, vitamin D binding protein (DBP) and albumin transport vitamin D and its metabolites in the bloodstream to the target organs [14,15]. Cytochrome P450 (CYP) enzymes are a superfamily of enzymes that are primarily responsible for the breakdown of xenobiotics, including medications, environmental pollutants, and natural health products [16,17]. However, CYP enzymes also catalyze reactions involved in the biosynthesis or breakdown of endogenous compounds such as hormones and vitamins. Select CYP enzymes are critical in the anabolism and catabolism of vitamin D, which undergoes CYP-mediated activation and inactivation reactions [18,19]. The physiological functions of vitamin D are carried out by its active metabolites through the activation of the vitamin D receptor (VDR). At the same time, CYP enzymes are involved in the breakdown of the active metabolites of vitamin D [20,21]. 

The endocrine functions of vitamin D specifically control a wide range of autonomous biological processes, such as bone metabolism and cell division and proliferation. There is increased evidence of lower vitamin D levels and corresponding vulnerability to different types of cancers like prostate, colon, and skin [22,23,24]. In light of this, the ingestion of vitamin D_3_ supplementation has exponentially grown over the last decade or so. Vitamin D and its active metabolites demonstrate antitumor properties by regulating cancer cell differentiation, growth, and death in in vitro and preclinical animal models [25]. With skin as the primary site of physiological vitamin D synthesis and a location of VDR expression, vitamin D homeostasis will likely have a profound effect on the development, treatment, and progression of melanoma. Indeed, vitamin D and its derivatives have anticancer effects against melanoma and may play a critical role in the prevention and treatment of melanoma [26,27,28,29]. Interestingly, low vitamin D levels have been proposed as one of the factors responsible for the development of melanoma. Similarly, combining vitamin D with synthetic anticancer drugs has been related to better treatment outcomes for melanoma. 

Vitamin D is a promising anticancer agent. Researchers have studied its potential anticancer actions in several cancers, including melanoma [7,26,30,31,32,33,34,35]. It is intriguing that low vitamin D is a potential cause of a cancer type that develops from excessive exposure to the sun, and it is well established that exposure to the sun facilitates vitamin D synthesis. This suggests that synthesis or ingestion of vitamin D is not the only factor determining its physiological levels, and different components of pharmacokinetics, especially metabolism, play a key role in vitamin D homeostasis. CYP enzymes are the primary driver of vitamin D bioactivation and catabolism. The objective of this review is to understand the role of vitamin D in melanoma and evaluate the involvement of CYP enzymes in modulating vitamin D action. Since CYP enzyme levels and functions are commonly affected by xenobiotics, CYP-mediated vitamin D metabolism can influence vitamin D actions in melanoma. 

## 2. Literature Search Strategy

The literature search for this narrative review was carried out utilizing specific keywords across prominent electronic databases until 1 February 2024. Variations of key terms, such as vitamin D, melanoma, deficiency, cytochrome P450, therapeutic prevention, dermal expression, metabolism, anticancer effects, vitamin D supplementation, and their combinations were considered. PubMed, Medline, and Google Scholar databases were searched. Original research articles in English were included, whereas conference abstracts and unpublished materials were excluded. Titles and abstracts were initially screened, followed by a full-text examination. The literature search and reconciliation of resources were independently carried out by two authors (S.D. and M.B.E.), with any discrepancies resolved through consensus.

## 3. Cytochrome P450-Mediated Vitamin D Metabolism

**Figure 1:**
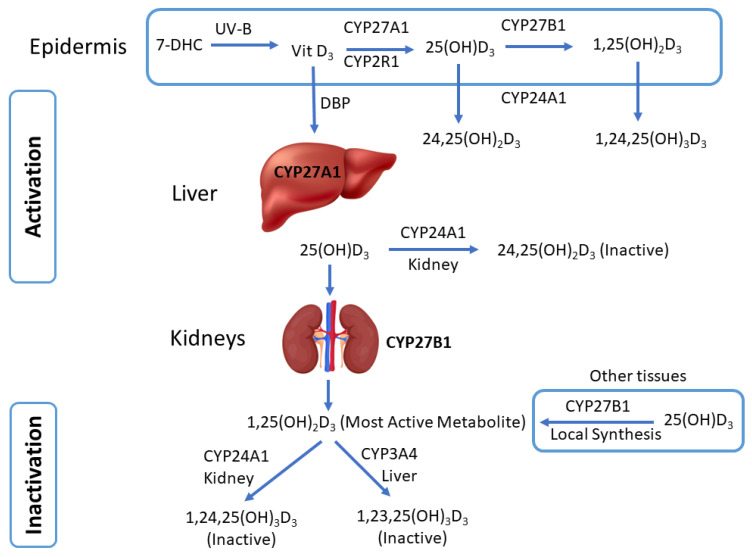
Cytochrome P450 (CYP)-mediated metabolism (anabolism/catabolism) of vitamin D.

Vitamin D functions as a hormone on numerous extraskeletal sites and exhibits an intricate multistep metabolism [36]. The parent vitamin D that first enters the human body or is synthesized is inactive and must undergo CYP-mediated activation via hydroxylation reactions to become active. Multiple CYP enzymes, including CYP27A1, CYP27B1, CYP2R1, CYP2J2, CYP24A1, and CYP3A4, are involved in the biotransformation of vitamin D, which are expressed in multiple organs, including the skin (Figure 1). Table 1 summarizes relevant information on the CYPs involved in vitamin D activation and inactivation reactions. The bioactivation of vitamin D_3_ is facilitated by 25- and 1α-hydroxylation reactions to form 1α,25-dihydroxyvitamin D_3_ [1α,25(OH)_2_D_3_] or calcitriol, the most active and potent VDR ligand [15]. In contrast, the inactivation steps of 1α,25(OH)_2_D_3_ involve 24- and 23-hydroxylation [20,37]. The epidermis layer containing keratinocytes can locally produce the active form of vitamin D and can also facilitate the inactivation of active vitamin D [38,39,40]. However, the liver and kidney are the primary sites of activation and inactivation after DBP transports the parent vitamin D to systemic organs [18,20,41]. The hepatic CYP enzymes hydroxylate vitamin D to produce 25-hydroxyvitamin D_3_ [25(OH)D_3_], which is the widely accepted clinical serum marker of vitamin D. Subsequently, DBP transports 25(OH)D_3_ to the kidneys for further hydroxylation to 1α,25(OH)_2_D_3_ [15]. CYP11A1 enzyme attacks the side chains and D-ring of 7-DHC and vitamin D_3_ to produce atypical vitamin D derivatives [19]. 

Calcitriol is the active and most potent form of vitamin D and is formed through two consecutive hydroxylation reactions. CYP27A1 is expressed in the liver, whereas CYP27B1 and CYP24A1 are primarily expressed in the kidneys. Similarly, for CYP2R1 and CYP3A4, the liver is the primary site of expression, along with their presence in dermal fibroblast [48]. Mitochondrial CYP27A1 is the major enzyme responsible for the C-25 hydroxylation of vitamin D_3_. In addition, microsomal CYP2R1 and CYP2J2/3 can also contribute to the formation of 25(OH)D_3_ [36]. In response to calcium homeostasis, CYP27B1, another mitochondrial enzyme, catalyzes the second hydroxylation step at C-1α in the bioactivation process to produce active calcitriol. Alternatively, calcitriol and other vitamin D_3_ derivatives are known to enhance CYP3A4 transcription via VDR [49]. Other cells throughout the body, including different cancer cells, can also synthesize calcitriol [50,51,52].

Inactivation of the active forms of vitamin D primarily occurs through the CYP24A1 enzyme in extrahepatic tissues, including the kidney and skin, whereas CYP3A4 is the major vitamin D inactivating enzyme in the liver [19,20,53,54,55]. CYP24A1 catalyzes multistep reactions of converting calcitriol to inactive lactone and calcitroic acid through the C-23 and C-24 pathways, respectively [37]. In contrast, CYP3A4 inactivates calcitriol via hydroxylating at C-23, C-24, and potentially at another carbon on the side chain [20]. It is worth recognizing that CYP3A4 is the major hepatic CYP enzyme, with majority of the drugs in the clinic as its substrate. Its expression and activities are known to be modulated by a diverse class of medications, natural products, and dietary substances. 

Vitamin D derivatives can undergo phase II conjugation reactions involving uridine diphosphate (UDP)-glucuronosyltransferases (UGT) and sulfotransferases (SULTs), though this area has not been explored comprehensively. Due to the presence of multiple hydroxyl groups in the vitamin D derivatives, they are suitable substrates of these conjugating enzymes. Calcitriol has been identified as a substrate for UGT1A4, UGT2B4, and UGT2B7 isoforms [56]. SULT2A1 catalyzes the sulfation reaction of several vitamin D_3_ derivatives, including 7-DHC, vitamin D_3_, 25(OH)D_3_, and calcitriol. In addition, calcitriol and 7-DHC are substrates of SULT1A1 and SULT2B1b, respectively [57]. 

## 4. Dermal Expression of Vitamin D-Related CYP Enzymes

Since the skin is the primary site of vitamin D synthesis, the dermal expression of CYP enzymes can play a critical role in the homeostasis of vitamin D (Table 2). Though extradermal organs have the highest expression of vitamin D-related CYP enzymes, it is important to recognize that CYP27A1, CYP27B1, and CYP24A1 are present in different layers of skin with all the three enzymes present in the epidermis (keratinocytes) and CYP27A1 and CYP24A1 expressed in the dermis (fibroblast) [38]. CYP11A1, CYP24A1, CYP27B1, CYP2R1, and CYP3A4 are expressed in WM98 and A375 human melanoma cell lines [58]. The 25-hydroxylase, 1α-hydroxylase, and 24-hydroxylase activities for vitamin D were modulated by vitamin D derivatives in MeWo melanoma cells but not in SkMel5 cells [59]. CYP27A1 and CYP27B1 are expressed in MeWo, SK-Mel28, SM, SK-Mel-5, SK-Mel-25, IGR, and MelJuso melanoma cell lines; however, the last four cell lines in this list are not responsive to 25(OH)D_3_ and calcitriol treatment [60]. Interestingly, calcitriol upregulated CYP24A1 expression by 7000-fold in MeWo, SK-Mel28, and SM cell lines [60]. CYP2R1 and CYP27A1, the enzymes responsible for C-25 hydroxylation of vitamin D, are expressed in dermal fibroblasts [48]. CYP27B1 was identified in human melanoma tumor tissue [61]. CYP27B1 expressions have been reported to be inversely correlated with the development of melanoma, and low or undetectable levels have been linked to poor prognosis [62,63]. 

CYP24A1, the most prominent catabolic enzyme, has been detected in tissues expressing VDR and plays a vital role in the local regulation of vitamin D activity [36]. Several human malignancies, including melanoma, have been shown to have higher CYP24A1 levels [55]. CYP24A1 protein levels are inversely linked with poor clinical outcomes in patients with melanoma [55]. Maintaining homeostasis of 1α,25(OH)_2_D_3_ is the principal function of 24-hydroxylase (CYP24A1) [26]. The CYP24A1-mediated inactivation of 1α,25(OH)_2_D_3_ reduces the antitumorigenic capacity of the VDR signaling pathway [55]. Similar to VDR and CYP27B1, absent or diminished CYP24A1 was determined to be correlated with shorter overall and disease-free survival [62]. Brozyna et al. also found that the expression of CYP24A1 was greatest in patients with benign nevi and localized melanoma, while patients with metastatic melanoma depicted lower levels. A clinical pathology study with melanoma patients found that the occurrence of ulceration, necrosis, nodular type, and amelanotic phenotypes was related to lower CYP24A1 levels. It has been hypothesized that overexpressing CYP24A1 increases tolerance to the antiproliferative effects of calcitriol [55]. Moreover, the suppression of CYP24A1 has been suggested as an adjuvant in vitamin D-based cancer treatments [55]. 

A study carried out by Slominski et al. found that melanoma progression lowered the expression of the VDR and CYP27B1, and melanization and melanogenesis had a detrimental effect on stage III disease survival as well as overall survival [29]. Immunohistochemistry study of the human uveal tract and uveal melanoma, which is a tumor of the iris, indicates that CYP27B1 and CYP24A1 are expressed in the ocular compartment [70]. Interestingly, the expression of CYP27B1 is downregulated in tumors that advance to metastasis compared to the ones that did not metastasize [71]. CYP27B1 has been found to activate vitamin D, which protects against cancer [44]. Normal skin typically has high levels of CYP27B1 expression (>10%); nevertheless, CYP27B1 enzymatic activity (0–5%) has been noted in aggressive forms of melanoma and non-melanoma skin malignancies, including basal cell carcinoma [44]. The absence of CYP27B1 is linked to both disease-free progression and shorter overall survival. Due to the notable reduction in VDR and CYP27B1 expression, researchers believe that the low levels of these proteins promote melanoma development because of the lack of antiproliferative action of vitamin D [71]. Based on these findings and their consistency with previous ovarian and colon cancer studies, it can be concluded that CYP27B1 expression may be used as a biomarker for cancer presence and severity.

## 5. Vitamin D Signaling in Melanoma

**Figure 2:**
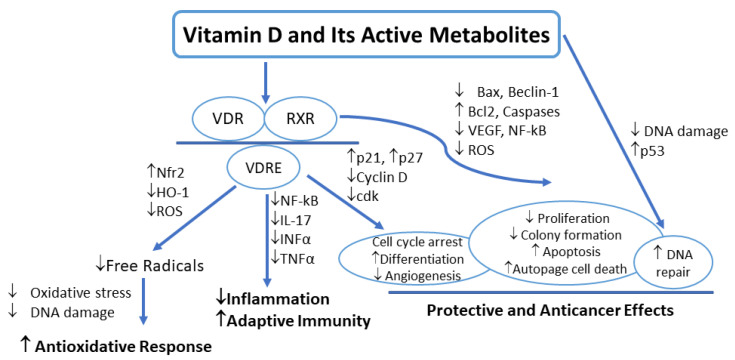
Anticancer signaling of vitamin D and its active metabolites.

The physiological activities of vitamin D are carried out through the activation of nuclear VDR by different vitamin D derivatives, followed by classical molecular signaling pathways (Figure 2). Though there are several natural and chemically synthesized vitamin D derivatives known to activate VDR, 1α,25(OH)_2_D_3_ is the most potent ligand of VDR [40]. Apart from the main function of maintaining calcium concentration and skeletal health, vitamin D mediates a multitude of anticancer effects, including antiproliferation, induction of apoptosis, and prodifferentiation [2,72]. Melanoma has been found to express VDR and respond to activities of active vitamin D, such as cell proliferation. This cell proliferation is regulated by signaling pathways where active vitamin D affects growth factors and proteins that control the cell cycle [2]. Slominski et al. state that melanin content may impact vitamin D signaling and its anticancer efficacy, which is consistent with the idea that blocking melanin formation would make melanoma cells more susceptible to antitumor therapy and prolong life. 1α,25(OH)_2_D_3_ suppressed cell growth in vitro transcriptional downregulation in human melanoma cell lines [25,36]. Wasiewicz et al. found that vitamin D signaling defects were correlated with worse prognosis or more advanced stages of melanoma [58]. As depicted in Figure 2, the metabolites of vitamin D carry out the physiological actions of vitamin D by activating the vitamin D receptor. When the VDR forms a heterodimer with retinoid X receptor, it binds to vitamin D response elements (VDREs) [58]. The resulting complex then downregulates the cytokines and signaling mediators, leading to the driving of anti-inflammatory and antioxidative actions. Furthermore, as concentrations vary, the complex that forms between VDR, RXR, and VDREs modulates the expression of specific cell cycle-controlling proteins, leading to cell cycle arrest. Activation of RXR and vitamin D and its metabolites has also been shown to exhibit anticancer activity such as antiproliferation, apoptosis, and DNA repair. To prevent oxidative DNA damage, active forms of vitamin D_3_ promote DNA repair by upregulating the expression of the p53 tumor suppressor gene [2].

Changes in vitamin D activation, local and systemic levels, and VDR signaling pathways can all lead to a loss of vitamin D anticancer protection, which may also promote the development of melanoma. Clinical data indicates that in cases of advanced melanoma, the VDR exhibits the lowest expression, whereas it is highest in normal skin. Patients with a clinical melanoma diagnosis and ulceration who have higher VDR expression seem to have better overall survival rates compared to those who have lower VDR expression since VDR expression is usually highest in normal cells [26]. The VDR is expressed in both melanocytes and keratinocytes, which respond to 1,25(OH)2D with decreased proliferation and enhanced differentiation. Major regulators of 1,25(OH)2D, such as interferon-γ (INFγ) and tumor necrosis factor-α (TNFα), are present in keratinocytes. Both TNFα and INFγ are activated by UVB, which increases the substrate through increased vitamin D production [40]. When tumors stop responding to vitamin D, it means that either the production of 1,25(OH)2D is suppressed or that posttranscriptional changes have eliminated VDR transcriptional activity [40]. The proliferation of both normal and pathological melanocytes, including melanoma cells, has been linked to modifications in the vitamin D endocrine system, which includes the VDR [72]. While signaling resulting from VDR activation regulates around 3% of the mammalian genome either directly or indirectly, alternative binding sites and receptors exist as well [2]. The VDR has the ability to carry out nongenomic functions, such as a variety of signaling pathways that could support topical 1,25(OH)2D protective effects [40]. An alternative binding pocket for 1α,25(OH)_2_D_3_ exists on the VDR, which induces prompt nongenomic reactions at the membrane level when occupied. Furthermore, as inverse agonists, active hydroxylated forms of vitamin D_3_ can interact with retinoic acid-related orphan receptors (RORs). They can thereby alter the physiological and molecular processes, such as cancer, that are controlled by these receptors. Inverse RORα/γ agonists may suppress tumor growth activity while also decreasing antitumor immunity [2].

When there is a deletion of CYP27B1 or VDR, defects in differentiation and increased proliferation of the epidermis are pronounced. In addition to the VDR, at least two other pathways modify vitamin D signaling and cause tumor growth in VDR null mice. The hedgehog (Hh) and Wnt/β-catenin pathways have both been implicated in this process [40]. The components of the Hh signaling pathway are overexpressed in the epidermis of animals lacking VDR. When Hh signaling is disrupted, basal cell carcinomic tumors will arise. An essential function of the Wnt/β-Catenin signaling system is to control the interactions between keratinocytes and melanocytes [39]. However, the exact function of this pathway in VDR function is still unknown. The Wnt/β-Catenin signaling pathway is unclear because certain research has linked its activation to a reduction in the growth of melanoma. When this pathway is inhibited, melanogenesis may be induced. Conversely, opposing studies have shown that Wnt/β-Catenin signaling is necessary for metastatic melanoma survival, and its inhibition leads to decreased proliferation, among other things [39]. Wnt signaling blocks the phosphorylation of serine within the β-Catenin exon. Due to this hindrance, β-Catenin is more readily available in the nucleus, where it interacts with the transcription factors to encourage the production of genes that are essential for cell division [40]. β-Catenin forms a junction complex with E-cadherin, which is increased by 1,25(OH)2D. When mutations or overexpression of the β-Catenin are present, skin tumors arise due to an increase in proliferation [40]. 

The long noncoding RNAs (LncRNAs) make up about 80% of the transcriptome and have been found to be master regulators of processes such as differentiation, embryonic pluripotency, and regulating histone modifications. Vitamin D signaling in the skin can also inhibit tumor growth by using LncRNAs. When abnormally expressed, LncRNAs are typically associated with cancer [40]. Various LncRNAs were noticeably elevated after studying the function of LncRNAs in VDR defense against skin cancers in mice. In VDR null mice, there is a shift in the expression of LncRNAs towards a more oncogenic profile [73]. In order to preserve the protective factors of VDR, the ratio of oncogenic to tumor suppressor LncRNAs must be maintained [40]. 

## 6. Vitamin D Deficiency and Melanoma

Studies have demonstrated a link between vitamin D deficiency and an increased risk of developing melanomas (Table 3). Lower plasma vitamin D levels are associated with the development of melanoma and/or reduced survival time in patients with metastatic melanoma [74,75,76,77,78,79,80]. Additionally, studies have also explored the relationship between dietary vitamin D supplements and the incidence of melanoma [11]. The findings indicated that while vitamin D and its metabolites have antiproliferative properties, there is no discernible pattern in the risk of melanoma associated with the length of time taken as a supplement, the daily dosage, or the average intake over ten years [11]. A new meta-analysis involving data from 211,230 patients showed that supplementation of vitamin D and blood 25(OH)D_3_ levels are modestly related to the risk of melanoma despite the uncertain relationship between melanoma risk and vitamin D intake. Nonetheless, a favorable association was discovered between vitamin D intake and an improved prognosis for melanoma patients [81]. Intake of vitamin D supplements was protective against the development of melanoma. Table 3 lists the studies exploring the association between vitamin D plasma levels and melanoma [6,63,82]. 

It is important to point out that there is an ongoing scientific debate between health societies on what the optimal vitamin D serum levels are. Optimal levels are not well defined. There is not yet a consensus on optimal levels and cut-off points. Different organizations have different definitions of vitamin D deficiency. Vitamin D levels are categorized as sufficient or optimal levels if the levels are more than 30 ng/mL, insufficient levels if between 12 and 20 ng/mL, and vitamin D deficiency if levels are less than 12–20 ng/mL [83]. However, there is still a lack of consensus on the appropriate vitamin D intake. Different recommendations have been made by various expert groups. The Institute of Medicine (IOM) advised a threshold value of 20 ng/mL with 600 IU per day to be adequate, whereas others suggest a benefit for a higher threshold of 25(OH)D levels ≥ 30 ng/mL. The recommendations from European and American Societies for Clinical Nutrition and Metabolism (ESPEN and ASPEN), the European Food Safety Authority, the National Institute for Health and Care Excellence (NICE), Australian high-priority, and the American Institute of Medicine guidelines recommend 1000 IU per day. In addition, the Endocrine Society recommended up to 2000 IU daily to reach adequate serum levels [84,85,86,87,88,89,90].

The retrospective review, cohort studies, cross-sectional studies, and randomized controlled trial studies explored the plasma levels of vitamin D in a population >50 years of age. Piotrowska et al. reported that vitamin D deficiency in melanoma was associated with poor prognosis [91]. In a 2020 study with approximately 1600 patients, the frequency of low vitamin D was significantly greater in individuals with melanoma compared to the melanoma-free subjects (OR, 2.12; 95% CI, 1.15–3.89) [92]. Furthermore, the literature also reports that greater vitamin D levels at diagnosis are associated with a better prognosis for melanoma and lower Breslow thickness (melanoma thickness) (*p* value = 0.002). Serum vitamin D levels are estimated to be considerably higher in patients with tumor thicknesses of less than 1 mm than in individuals with tumor thicknesses greater than 1 mm. The fact that individuals with melanoma with low vitamin D levels had much greater death rates than those with higher vitamin D levels is not surprising, considering these findings [92]. These findings are coherent with other studies conducted across the globe [3,39,93]. Recent research also claims that lower vitamin D (25 nmol/L) reduces the survival time in patients suffering from melanoma [39]. Similarly, Lombardo et al. (2021) reported that vitamin D deficiency was correlated with a higher risk of developing more advanced melanoma tumors [94]. 

**Table 3:** Examples of clinical studies on vitamin D levels and melanoma risk.

Author	Study Design	Sample Size	Location	Study Population	Duration of Follow Up	Mean Age	Objective	Intervention	Study Findings
Asgari et al., 2009 [11]	Cohort study	Male: 37,382, Female: 40,337	USA	Men and women from western Washington 50–76 years	10 years	62 years (range, 50–76 years	To examine the association between vitamin D intake and melanoma risk	Average intake in µg/day over 10 years from multi- vitamin and individual supplement sources	No association between total intake of vitamin D and melanoma risk
De Smedt et al., 2022 [82]	Multicentre randomized double-blind placebo-controlled phase III trial	Male: 78, Female: 77	Belgium, Hungary	Patients with stage IB to III Cutaneous melanoma (according to the 7th AJCC staging), age 18–80	NA	Group A 56 (47–66), Group B 54 (45–64)	To investigate the connection between 25(OH)D_3_ levels, tumor-node-metastasis (TNM) stage and histopathological parameters	25(OH)D_3_ plasma levels	Low levels of 25(OH)D_3_ were associated with a worse outcome in patients with melanoma
Liyanage et al., 2020 [6]	Mendelian randomization study	Cases: 12,874 and Controls: 23,203	Australia	Summary data from a large genome-wide association study	NA	NA	To investigate a causal association between genetically predicted 25(OH)D_3_ concentrations and melanoma using a Mendelian randomization (MR) approach	Genetically predicted 25(OH)D_3_ concentrations	Low levels of 25(OH)D_3_ were not associated with the risk of melanoma [(OR) 1.06, 95% confidence interval (CI) 0.95–1.19]
Reichrath et al., 2022 [63]	Prospective, comparative, non-interventional side-by-side study	83	Germany	Treatment with CTLA-4i, PD-1i, or BRAFi/MEKi for histologically and clinically confirmed advanced melanoma	2013–until death	63.43 years	To investigate whether vitamin D status is predictive of efficacy and safety in patients treated with immune checkpoint or BRAF/MEK inhibitors	Vitamin D levels in plasma	Vitamin D deficiency was associated with poor clinical outcomes in patients treated for metastasized melanoma with BRAF/MEK inhibitors or immunotherapy
Befon et al., 2020 [93]	Prospective cohort study	105, Male: 46, Female: 53	Greece	Consecutive patients diagnosed with primary invasive CMM of any stage were enrolled.	42–78 months	≤40 years: 31 ± 31.31	Association of serum total 25(OH)D_3_ levels at melanoma diagnosis with known risk and prognostic factors for CMM	Serum 25-hydroxy vitamin D levels of melanoma patients	Low levels of 25(OH)D_3_ were found in Greek cohort of melanoma patients vs. healthy controls
Stenehjem et al., 2020 [5]	Case-control study	1416, Case: 708, Control: 708	Norway	Cases were histologically verified melanomas < 75 years of age	Year 1972–2003 to 31 December 2009	Cases: 42 (22–67), Control: 42 (22–67	To study 25(OH)D_3_ and melanoma risk in the population-based Janus Serum Bank Cohort	Concentrations of 25(OH)D_3_ were measured during 2016–2017	Adequate vitamin D levels were associated with a reduced risk of melanoma
Fearfield et al., 2019 [74]	Retrospective study	104 patients	UK	Patients with primary and metastatic melanoma	May 2016 to October 2017.	Range: 23–85 years	To determine levels of 25(OH)D_3_ in patients with primary and metastatic melanoma	Plasma levels of vitamin D	80% of patients with melanoma had vitamin D deficiency
Timerman et al., 2017 [75]	retrospective, single-center study	252, Male: 144, Female: 108	USA	Patients with melanoma and at least one serum 25(OH)D_3_ measurement within one year after the diagnosis	January 2007 to June 2013	55.4 ± 14.7	The initial serum 25(OH)D_3_ concentrations and the change in 25(OH)D_3_ levels were associated with melanoma prognosis	Serum 25(OH)D_3_ concentrations	Vitamin D deficiency was associated with a worse prognosis in patients with metastatic melanoma
Lombardo et al., 2021 [94]	retrospective, single-center study	154, Male: 78, Female: 76	Italy	Patients with melanoma with low Vitamin D serum levels. Control groups with a negative melanoma history	3-year period 2016 to 2019	59.7 ± 15.5	To study the association between vitamin D serum levels and germane prognostic factors/pathological parameters in melanoma	25(OH)D_3_ serum levels were measured during 2016–2019	Vitamin D deficiency is a possible predisposing factor for the development of melanoma
Moreno-Arrones et al.,2019 [78]	cross-sectional, single-center study	204 patients	Spain	Patients with primary invasive melanoma	2013 to 2017	-	To investigate the association between vitamin D serum levels and pathological parameters in melanoma	Serum 25(OH)D_3_ concentrations	There was a significant association between low vitamin D levels and an increased risk of developing aggressive melanoma
Bade et al., 2014 [76]	retrospective, single-center study	324 melanoma patients, 141 healthy controls	Germany	Melanoma patients	February 2000 and April 2004	56.3	To study the association between low vitamin D serum levels and risk for melanoma prognosis	Serum 25(OH)D_3_ concentrations	Low serum 25(OH)D_3_ concentrations were associated with poor outcomes in melanoma patients, predicting risk and prognosis of melanoma
Gambichler et al., 2013 [77]	Prospective study	764 patients	Germany	Malignant melanoma	-	-	To study the association between vitamin D serum levels and clinical parameters in melanoma patients	Serum 25(OH)D_3_ concentrations	Low levels of vitamin D were associated with advanced tumor stage
Nurnberg et al., 2009 [79]	Prospective study	205 Patients, 141 health controls	Germany	Stage 4 melanoma patients	December 1997 to March 2007	-	To study the association between vitamin D levels and clinical and histopathological parameters among melanoma patients	Serum 25(OH)D_3_ concentrations	Patients with metastasized melanoma stage 4 had significantly lower vitamin D levels
Moro et al., 2022 [80]	Retrospective, observational, longitudinal	286 patients with history with melanoma;	Spain	Patients with a histological melanoma	Follow-up period of 39.4 months	-	To study the prognostic value of vitamin D serum levels in melanoma patients	Serum 25(OH)D_3_ concentrations	Low vitamin D levels were associated with ulceration in melanoma

Various studies have demonstrated an association between obesity and low 25(OH)D_3_ levels. It is thought that because vitamin D is fat-soluble and has a reduced bioavailability, overweight people are disposed to have lower vitamin D levels than people of normal weight. Therefore, some studies propose to use body mass index (BMI) as a possible confounding factor in analyzing the relationship between melanoma and obesity. Although the relationship between weight and BMI and the occurrence of melanoma is not entirely known, it is thought to be less important than exposure to UV radiation [5]. In a similar study comparing individuals with cutaneous melanoma to those with normal BMI levels (≥18.5–<25 kg/m^2^), it was discovered that a BMI of ≥25–30 kg/m^2^ or >30 kg/m^2^ was a critical reason for 25(OH)D_3_ levels < 20 ng/mL. This negative association between 25(OH)D_3_ levels and BMI is consistent among adults and children from varying ethnic backgrounds and geographic areas [82].

Globally, fair-skinned populations are experiencing an increase in the incidence and fatality rates of cutaneous melanoma [5]. In Northern Europe, serum 25(OH)D_3_ levels tend to be higher than those in Southern Europe. Comparable results are observed in Western and Eastern Europe, with greater 25(OH)D_3_ levels in the former region. The different skin phototypes found in European populations are thought to be the cause of this variation in 25(OH)D_3_ levels [93]. When groups have increased pigmentation, there tends to be a safeguard against the negative effects of UV radiation, UV-B-induced sunburn, DNA damage, and skin carcinogenesis [73]. When compared to people with lighter characteristics, this conclusion is illustrated in those with light complexion and dark hair. Individuals with cutaneous melanoma who had light skin and dark-brown or black hair compared to those with light skin and blonde or light brown hair had a decreased chance of plasma 25(OH)D_3_ levels below 20 ng/mL (OR 0.47, 95% CI 0.29–0.77, *p* = 0.003) [82]. Low vitamin D levels are concerning as <20 ng/mL or less of blood total 25(OH)D_3_ is considered deficient [93]. Approximately 21–29 ng/mL is considered insufficient, while greater than 30 ng/mL is considered adequate [93]. 

The relationships between sun exposure, vitamin D levels, and melanoma risks are complex and not fully understood. It is known that increasing sun exposure maximizes vitamin D production and boosts serum vitamin D levels while minimizing skin cancer risk [95]. However, studies have shown that people with adequate exposure have been reported to have low vitamin D levels [96]. The antioxidant actions of vitamin D in cancer are well established (Figure 2). Low vitamin D levels were associated with an increase in oxidative stress marks in many human diseases [97,98]. However, vitamin D supplementation reduces oxidative stress [99,100]. Studies have linked melanoma with an increase in oxidative stress [101,102]. It has been demonstrated that patients with NMSC and melanoma presented with an overproduction of free radicals [103]. Karampinis et al. studied the role of vitamin D levels in non-melanoma skin cancer (NMSC) patients. The results revealed that the majority of the included NMSC patients had low vitamin D levels and were presented with higher levels of systematic oxidative stress markers. However, the higher vitamin D levels were positively correlated with lower oxidative stress markers [104].

Another study reported by Reichrath et al. in melanoma patients found that lower vitamin D levels (25(OH)D_3_ s.c. 10 ng/mL) were correlated with worse overall survival (OS) and progress-free survival (PFS), increased tumor load, and risk of adverse events [63]. Although all these results validate the correlation between vitamin D and clinical outcomes, including a connection between a smaller Breslow tumor thickness and a higher 25(OH)D_3_ level, published reports have shown inconsistent results [5,26,82,92]. The competing roles of UVR in the etiology of cancer and vitamin D production mean that, despite ongoing studies on melanoma, the causal link between vitamin D and melanoma remains unclear [6]. Furthermore, the precise role of vitamin D in the development of melanoma remains unknown. However, due to the complex connection between melanoma and vitamin D levels, along with its resulting inconsistent outcomes, it is reasonable to assume that correlation does not amount to causation [92]. In fact, a Mendelian randomization (MR) study was conducted in 2020 to ascertain the plausibility of a causal relationship between vitamin D and the incidence of melanoma. While researchers found no evidence of a causal link to melanoma, both direct and reverse causality cannot be definitively ruled out. According to reverse causality, the cancer diagnosis itself could be the reason for decreased 25(OH)D_3_ levels [5,63]. As researchers continue to explore the pathogenesis behind melanoma, it may be inferred that vitamin D is more significant in slowing the progression of melanoma than in preventing its development [92]. 

## 7. Factors Affecting CYP-Mediated Vitamin D Metabolism and Vitamin D Effects

The modulation of CYP enzymes that facilitate vitamin D activation or inactivation can have a significant effect on its plasma concentration. Activation of vitamin D occurs in the liver and kidneys by sequential reactions of 25-hydroxylation (CYP27A1, CYP2R1) to form 25(OH)D_3_ (liver); further, it undergoes another 1α hydroxylation (CYP27B1) to form 1α,25(OH)_2_D_3_ (kidneys). In contrast, 24-hydroxylation (CYP24A1) of the 25(OH)D_3_ or 1α,25(OH)_2_D_3_ in the kidney plays a major in the catabolism or inactivation of vitamin D [21,105]. Vitamin D-metabolizing CYP enzymes are regulated via multiple receptors, including VDR, pregnane X receptor (PXR), and steroid and xenobiotic receptor (SXR) [18,21,106,107,108]. Factors that influence the activation of these receptors and the regulation/function of CYP enzymes catalyzing vitamin D metabolism can eventually affect vitamin D levels and their actions. The polymorphism of VDR and vitamin D-related CYPs can influence vitamin D levels and actions. Similarly, different disease conditions can also affect vitamin D activation. 

### 7.1. Xenobiotics as Inducers of Vitamin D Metabolism

CYP enzymes that facilitate vitamin D anabolism or catabolism are susceptible to induction by numerous medications and natural health products. Several studies have investigated the impact of CYP induction on tissue-specific vitamin D metabolism and plasma levels, as those enzymes are expressed in a variety of tissues [109]. Multiple receptors, such as the SXR and PXR, are involved in the regulation of CYP enzymes. A large number of drug classes (e.g., antiepileptics, St. John’s wort, Kava Kava, antibiotics, and HIV drugs) work as PXR ligands and can increase the breakdown of calcitriol through CYP3A4 or CYP24A1 induction. Antiretrovirals such as ritonavir and saquinavir are known to cause vitamin D deficiency, followed by other bone-related problems, through induction of CYP-mediated active vitamin D metabolism [110]. Antiretroviral medications efavirenz, stavudine, and ritonavir have been shown to upregulate CYP24A1, leading to a lower calcitriol level [109]. Drug-induced osteomalacia has been linked to increased CYP24A1 in the liver and gut via phenobarbital-and rifampicin-mediated activation of human PXR and better metabolic clearance of 1α,25(OH)_2_D_3_ and 25(OH)D_3_ [18]. Activation of SXR has been found to reduce the VDR-mediated CYP24A1 promotor function [21]. SXR-mediated induction of CYP24A1 leads to enhancement of vitamin D degradation, diminishing the biological effects of vitamin D [21]. 

Similarly, antituberculosis treatments such as isoniazid (INH) and rifampicin (RIH) induce the expression of CYP27A1 in hepatocytes. The data from coadministration of the anti-TB medications in mice suggests that RIF and INH have a negative effect on vitamin D actions. RIF induces 24-hydroxylation in the kidney and accelerates vitamin D breakdown. Sheng et al., however, found that mice given single doses of RIF or combined RIF and INH exhibited increased 25-hydroxylase enzyme in the liver, which counteracted drug-mediated vitamin D catabolism and elevated serum 25(OH)D_3_ [111]. While administration of each drug individually did not affect CYP2R1 transcription, combined treatment with 100 mg/kg RIF and 50 mg/kg INH also showed a considerable increase in CYP2R1 mRNA expression [111].

Vitamin D levels can be affected by drugs and dietary supplements that work as CYP3A4 inhibitors or inducers [112]. Dexamethasone, a common anti-inflammatory glucocorticoid, is often administered before chemotherapy for advanced malignancies. It was found to have a significant negative impact on 1α,25(OH)_2_D_3_ levels, calcium absorption, and bone metabolism in a study with infant pigs. Considering these findings, a study assessing the effects of prednisolone and dexamethasone on mouse hepatic biotransformation was carried out. Compared to prednisolone, the dexamethasone-treated animals showed a greater amount of 1α,25(OH)_2_D_3_ breakdown by CYP3A isoforms. The biological effects of vitamin D3 may be impaired as a result of this enhanced hepatic breakdown [20]. Antiepileptic medications such as phenobarbital and phenytoin upregulate CYP3A4 and CYP24AA1 expression in cell culture experiments, leading to a purported decrease in serum 25(OH)D_3_ levels and enhancing the clearance of vitamin D metabolites [113]. Antiepileptic medications like carbamazepine or phenytoin have been shown to induce CYP3A4, leading to reduced benefits of vitamin D and the potential development of osteomalacia [114,115,116]. Similarly, CYP3A4-mediated 4-hydroxylation of 25(OH)D_3_ was increased by rifampin [114]. Autoinduction of CYP3A4-mediated metabolism of active vitamin D is another phenomenon that can lower vitamin D levels [41,107]. The effect of a diverse class of drugs on vitamin D metabolism has been reviewed elsewhere [110,117]. 

### 7.2. Xenobiotics as Inhibitors of Vitamin D Metabolism

The inhibitors of vitamin D metabolism-related CYP enzymes can influence the endogenous levels of parent and active vitamin D derivatives. The modulators can be classified as either inhibitory towards the bioactivation or the inactivation step. Antiretroviral medications efavirenz, stavudine, and ritonavir have been shown to downregulate CYP2R1, leading to a 30–45% reduction in cellular levels of calcitriol [109]. Several studies have reported antiretroviral therapy-mediated disruption of vitamin D activation, which could affect the efficiency of the skin as the first line of immune defense [109]. Interestingly, numerous studies have reported the association between low vitamin D levels and the risk of developing COVID-19 infection and its associated severe symptoms [118,119]. Ellfolk et al. showed that medications such as phenobarbital (antiepileptic) and efavirenz (anti-HIV) downregulated CYP2R1 expression; however, CYP27A1 expression was not affected by these drugs [48]. In contrast, cyclosporine has been reported to be an inhibitor of CYP27A1 in studies with cell culture and animal models [120]. Protease inhibitors (ritonavir, indinavir, and nelfinavir) block the activation of vitamin D by inhibiting the 25-hydroxylation and 1α-hydroxylation reactions [121]. 

A diverse class of medications, e.g., ritonavir, ketoconazole, clarithromycin, tamoxifen, and docetaxel, were able to inhibit hydroxylation-mediated deactivation of calcitriol [20]. Several medications have been reported to inhibit CYP24A1 and CYP3A4, the enzymes responsible for the breakdown of the active vitamin D. Ly et al. reported that 24-hydroxylation of calcitriol is blocked by liarozole [122]. Similarly, ketoconazole, either alone or in combination with tetralone or dexamethasone, can inhibit CYP24A1 activity [105,123]. Considering the flexible active site of CYP3A4, several drugs and natural products can inhibit the CYP3A4-mediated breakdown of calcitriol. Ketoconazole, a potent inhibitor of CYP3A, was shown to inhibit 1α,25(OH)_2_D_3_ hydroxylation in vitro in nanomolar concentration [20,116]. Ginsenosides such as aPPD and aPPT can block the CYP3A4-mediated hydroxylation of calcitriol [124,125]. Similarly, abiraterone, a prostate cancer treatment, can exert inhibitory effects on vitamin D catabolism [126]. Troleandomycin and azamulin, known potent inhibitors of CYP3A4, inhibited the CYPA4-mediated metabolism of 20(OH)D_3_, a vitamin D derivative with a biological efficacy similar to calcitriol [127]. 

### 7.3. Comorbidities

The presence of different diseases is another factor that can modify the vitamin D levels and their effects on melanoma. Researchers discovered a higher frequency of osteomalacia, severe vitamin D insufficiency, and low bone mineral density in individuals receiving HIV treatment. The combined use of antiretroviral medications, such as efavirenz, ritonavir, and stavudine, is thought to be the cause of this impact on bone and vitamin D levels [109]. It is believed that these drugs disrupt vitamin D homeostasis via mechanisms that alter gene expression. Protease inhibitors (PI), on the other hand, decreased the formation of 1α,25(OH)_2_D_3_ in monocyte-macrophage cell lines after HAART treatment [121]. The inhibition of 1α,25(OH)_2_D_3_ induced by PI may have a role in the demineralization of bone induced by PI-based therapy [36]. Obesity and type 2 diabetes are two metabolic disorders linked to a decrease in 25(OH)D_3_ levels. While many researchers believe that vitamin D levels are low due to storage in adipose tissue, another ongoing theory suggests that a reduction in CYP2R1 activity may be the source. Researchers looked at the obese animal models that were fed high-fat diets to verify this theory. CYP2R1 mRNA was shown to be substantially reduced in these obese mice than in lean animals [36].

### 7.4. Polymorphism

The endogenous vitamin D levels and their actions can be affected by polymorphisms in the gene encoding vitamin D metabolism-related CYP enzymes and VDR. Single nucleotide polymorphisms (SNPs) have been identified as frequent genetic risk factors for melanoma risk [53]. Studies have shown that VDR single nucleotide polymorphisms have been linked to poor survival rates in patients with melanoma [3,44]. The A allele of the VDR gene was shown to be substantially correlated with both metastasis and melanoma susceptibility in a study that examined the significance of VDR polymorphisms in melanoma risk [53]. Researchers examined multiple VDR gene polymorphisms in a study involving over 3600 individuals to identify that eight polymorphisms are statistically significantly associated with the risk of developing subsequent new primary melanomas [53]. Breslow tumor thickness is one of the most significant prognostic factors used for patients with cutaneous melanoma. The VDR polymorphism rs1544410 has been linked to an elevated risk of melanoma and an increased thickness of Breslow tumors. Reduced survival and the possibility of metastasis increases with tumor thickness [53]. The FokI (C/T-rs2228570) and BsmI (rs1544410) mutations are among the several SNPs found in the VDR gene that have been widely used in melanoma research. The FokI polymorphism results in a longer and less active VDR protein by generating a novel start codon upstream from the typical start codon. Conversely, the BsmI polymorphism limits the length of the fragment and causes silent changes that may change the VDR gene transcription and the stability of the mRNA [39]. 

Similarly, polymorphism of CYP enzymes involved in vitamin D metabolism can influence vitamin D levels. The polymorphisms in CYP24A1 and CYP27B1 were linked to prostate and colon cancer, respectively [39]. Despite there being few studies involving CYP-related polymorphisms, a variant of rs4646536 in CYP27B1 was identified to have probable relevance in carcinogenesis [39]. The loss of CYP27B1 activity led to poorer outcomes in melanoma [3]. Likewise, the CYP3A4 I301T mutation shows greater activity for the inactivation of vitamin D metabolites, resulting in decreased vitamin D levels. Following the loss of CYP3A4 function, a link between the CYP3A4 rs2242480TT variant and elevated 1α,25(OH)_2_D_3_ levels were observed among an elderly study population in Singapore. Certain polymorphisms were shown to increase cancer risks among various ethnic groups. For instance, it is believed that Africans with the CYP3A4*1B mutation have a higher risk of developing cancer, with small cell lung and prostate cancer being the most prevalent types [112]. 

## 8. Therapeutic Prevention of Melanoma by Vitamin D Supplementation and Modulation of CYPs

The development and progression of melanoma may be affected by VDR expression and signaling [112]. Vitamin D may play a vital role in the prevention of cancer because of its antiproliferative properties [128]. The prevention and treatment of melanoma can be influenced by vitamin D signaling in both local or systemic effects on vitamin D activation and inactivation. Over the last decade, substantial attention has been given to the role of vitamin D in preventing cancers, particularly in reducing the risk of melanoma [4,58]. Regarding epidemiological studies, the function of systemic vitamin D in melanoma patients is undetermined due to ambiguous evidence. Though there are convincing clinical reports about the effect of low vitamin D serum levels on the risk of development and progression of melanomas, the causal relationship is not confirmatory [6,81,92,129,130,131]. On the contrary, experimental evidence amply supports the anti-melanoma capabilities of vitamin D and its derivatives [11,26]. Additionally, molecular and clinicopathological reports have shown an association between deficiencies in vitamin D signaling and melanoma development and prognosis. This indicates that vitamin D signaling may be significant in treating melanoma. 

Supplementation with vitamin D has been shown to reduce the development of advanced cancers and mortality [132]. Meta-analyses of randomized clinical trials (RCTs) of vitamin D have shown that supplementation with vitamin D lowers cancer-related mortality rates [133]. Improving vitamin D signaling could be one of the strategies to prevent melanoma. Vitamin D signaling may be augmented by either increasing or correcting low plasma vitamin D levels by influencing the CYP-mediated metabolism of active vitamin D or through dietary supplementation. CYP24A1 and CYP3A4 are two key enzymes that are accountable for the breakdown of 1α,25(OH)_2_D_3_, the most active form of vitamin D. Higher CYP24A1 levels are indicators of poor prognosis of melanoma [55]. It is plausible that inhibition of CYP24A1 can prevent the development of melanoma. In other cancer types (e.g., prostate cancer and breast cancer), CYP24A1 or CYP3A4 inhibitors blocked the degradation of active vitamin D_3_ and improved the treatment outcomes in preclinical models or clinical studies [105,134,135,136,137]. Thus, CYP enzymes can be a viable target in the prevention or treatment of melanoma. 

In a study observing the consequences of targeting the vitamin D endocrine system in malignant skin diseases, vitamin supplementation was explored as a therapeutic option. The intake of 1000 mg of calcium and 400 IU of vitamin D daily by women with a history of non-melanoma skin cancer decreased their risk of developing melanoma compared to the placebo group. These findings suggest that future therapy options for at-risk groups, such as those with a present or past diagnosis of non-melanoma skin cancer, may include vitamin D and calcium supplements [72]. Slominski et al. (2015) suggested that higher doses of vitamin D (50,000 units/week or 10,000 units/day) can be administered to provide adjuvant (stage III/IV) or preventive benefits (stage I/II) [138]. The preventive effects of vitamin D supplementation were further evidenced in a randomized controlled study where a 57% lowering of melanoma development was observed in postmenopausal women who took 400 IU and 1000 mg of calcium daily [139]. Smedt et al. (2022) reported that melanoma patients without vitamin D supplementation were at a higher risk of developing low 25(OH)D_3_ levels [82]. Though the risk of low vitamin D levels and the development or progression of melanoma is relatively well accepted, the benefits of vitamin D supplementation in preventing melanoma development need more evidence. 

## 9. Conclusions

Here, we reviewed the available evidence on the role of vitamin D in melanoma and CYP-mediated metabolism, which can potentially affect the actions of vitamin D in melanoma. There is mounting evidence that vitamin D supplementation has the potential to exhibit VDR-mediated anticancer properties in melanoma. Vitamin D-related CYP enzymes are present in the epidermis (keratinocytes) and dermis (fibroblast), which are the primary sites of vitamin D synthesis. Selective targeting of catabolic CYP enzymes appears to be promising in the prevention of melanoma. Future studies on the combination of vitamin D dietary supplementation or vitamin D-related CYP modulators and chemotherapy should be undertaken to further our understanding of the prevention and treatment of melanoma. The influence of CYP polymorphic forms on the development and progression of melanoma is an area that needs close attention. The findings and limitations from past vitamin D studies should be considered in designing future clinical studies. 

## Figures and Tables

**Table 1:** Properties of cytochrome P450 enzymes relevant to vitamin D.

CYP	Tissue Localization	Substrate	Role (Anabolism/Catabolism)	Tissue Expression	Polymorphic (Yes/No)	Refs.
CYP11A1	Mitochondria	D_3_	Catabolism	Brain, GI tract, Skin	Yes	[2,42,43]
CYP24A1	Mitochondria	Calcitriol 25(OH)D_3_ 1α,24,25(OH)_3_D_3_ 1α,23,25(OH)_3_D_3_	Catabolism Hydroxylation	Kidney, Intestine, Skin	Yes	[15,37,44]
CYP27A1	Mitochondria	D_3_ 1α(OH)D_3_	Anabolism and Catabolism Pharmacological Hydroxylation	Liver, Skin, Macrophage	Yes	[37,44,45,46]
CYP27B1	Mitochondria	25(OH)D_3_	Anabolism and Catabolism Hydroxylation	Kidney, Skin	Yes	[15,37,44]
CYP2R1	Microsomes	D_3_	Physiological Hydroxylation	Liver, Skin	Yes	[37,46,47]
CYP3A4	Microsomes	Calcitriol D_3_	Hydroxylation	Liver, Intestine, Skin	Yes	[37,46]
CYP2D25	Microsomes	D_3_	Hydroxylation	Liver, Kidney	No	[46]
CYP2J2/3	Microsomes	D_3_	Hydroxylation Catabolism	Liver, Heart, Placenta, Brain	Yes	[36,46]

**Table 2:** Effects of vitamin D and its derivatives on dermal tissues and cell lines.

Vitamin D	Model	Signaling Proteins	Significance	Refs.
1α,25(OH)_2_D_3_	-Human melanoma cell lines -A375 -ME18 -MeWo -RPMI 7951 -SK Mel 28 -SKMEL-188 -WM35 -WM1341	-increase in Bcl-2 and Bcl-X (antiapoptotic) -decrease in BAX, GOS2, DAP-3, FADD, and caspases (proapoptotic) -upregulation of beclin-1	prodifferentiation	[2,58]
1,25,26(OH)_3_D_3_	-malignant melanoma MM96 cells -mouse B16 hamster Bomirski melanomas	-increasing the expression of TGFβ -blockade of epithelial–mesenchymal transition (EMT) -blocking of MMP-2 and MMP-9 secretion	suppressed proliferation	[2]
21(OH)D	-SKMEL-188 -hamster Ab -AbC1 melanoma lines	-decreased expression of VDR, RXR, PDIA3, and CYP2R1	antiproliferative activity	[2,58]
20(OH)D_3_	-in vitro assays -melanoma cells -melanocytes -hamster melanoma	-inhibition of NFκβ activity -CYP24A1	enhanced anti-melanoma activity	[2]
20(OH)D_2_	-in vitro -malignant cells -melanocytes -hamster melanoma	VDR-mediated	antiproliferative activity	[2]
20,23(OH)_2_D	-in vitro assays -malignant cells -melanocytes -hamster melanoma	-VDR-mediated -Albumin	antiproliferative	[2,58]
20,23(OH)_2_D_3_	malignant cells	-VDR-mediated	antiproliferative	[2]
20,24(OH)_2_D_3_	-in vitro -immunostaining of melanomas	-CYP24A1	anti-melanoma activity	[2]
20,25(OH)_2_D_3_	-human melanoma cells	-CYP24A1	inhibits proliferation	[2,55]
1,25(OH)_2_D_3_ 25(OH)D_3_	-human melanoma cell lines -MeWo -MeWo (EB1089) -SkMeI28 -SkMeI28 -(EB1089)2.05 -SkMeI25 -IGR -MeIJuso	-VDR-mediated -CYP24A1	antiproliferative	[60]
1,25(OH)_2_D_3_	-human melanoma cell lines -MeWo -SkMeI5	VDR-mediated -CYP24A1	inhibits proliferation	[59]
1,25(OH)_2_D_3_	-RPMI 7951 (high VDR) -SK-MEL-28 (low VDR)			[64]
1,25(OH)_2_D_3_	-B16-F10	-increase in cleaved caspase-3, caspase 8, caspase 9, Beclin, and PARP	antiproliferative	[65]
1,25(OH)_2_D_3_	-human melanoma cell lines -MeWo -WM1341	-VDR-mediated	induce apoptosis	[66]
1,25(OH)_2_D_3_	-human melanoma cell lines-G-361/A3	-VDR-mediated -nuclear factor KB (NF-KB)	anti-inflammatory action	[67]
1,25(OH)_2_D_3_	-human melanoma cell line	-blocks the formation of the sphingolipid degradation product sphingosine 1-phosphate (S1P)	induce apoptosis	[68]
1,25(OH)_2_D_3_	-CRL-1619	-inhibition of oxidative DNA/RNA damage	anti-inflammatory action	[69]

## Data Availability

Not applicable.
